# New Insights on Self-Employment of Older Adults in the United States

**DOI:** 10.1093/geroni/igaa057.1490

**Published:** 2020-12-16

**Authors:** Joelle Abramowitz

**Affiliations:** University of Michigan, Ann Arbor, Michigan, United States

## Abstract

This work examines the nature of self-employment arrangements of older adults in the United States. Many people engage in self-employment - in the 2016 Health and Retirement Study (HRS), 20 percent of respondents working for pay reported being self-employed - yet there exists a dearth of data on these arrangements. This lack of data prevents consideration of important questions relevant to employment, inequality, and policy. Who works in different types of self-employment? What resources facilitate some individuals obtaining higher quality self-employment arrangements? To what extent does the income from different types of arrangements keep people out of poverty? Are different types of arrangements associated with individuals being happier and having more job satisfaction? This work leverages novel restricted-access data collected in the HRS in 2016 on the employer names and locations for individuals reporting self-employment along with respondent narratives on industry and type of work to classify self-employment reports into three entrepreneurial roles (own/run; manage; independent) across 14 different types of work. Using the breadth of information collected in the HRS and linkage to administrative records, this work then presents differences in characteristics, such as demographics, income, wealth, savings, health insurance coverage, home ownership, health status, and expectations of working longer, associated with different classifications of self-employment. Exploring these questions provides unique insights into the changing nature of work and the transition to retirement relevant to policy considerations across the health, insurance, and retirement income dimensions, among others.

